# Histone H3K27 Methylation Perturbs Transcriptional Robustness and Underpins Dispensability of Highly Conserved Genes in Fungi

**DOI:** 10.1093/molbev/msab323

**Published:** 2021-11-09

**Authors:** Sabina Moser Tralamazza, Leen Nanchira Abraham, Claudia Sarai Reyes-Avila, Benedito Corrêa, Daniel Croll

**Affiliations:** 1 Laboratory of Evolutionary Genetics, Institute of Biology, University of Neuchatel, Neuchatel, Switzerland; 2 Department of Microbiology, Institute of Biomedical Sciences, University of Sao Paulo, Sao Paulo, Brazil

**Keywords:** histone methylation, gene conservation, fungi, *Fusarium*, expression robustness, comparative genomics

## Abstract

Epigenetic modifications are key regulators of gene expression and underpin genome integrity. Yet, how epigenetic changes affect the evolution and transcriptional robustness of genes remains largely unknown. Here, we show how the repressive histone mark H3K27me3 underpins the trajectory of highly conserved genes in fungi. We first performed transcriptomic profiling on closely related species of the plant pathogen *Fusarium graminearum* species complex. We determined transcriptional responsiveness of genes across environmental conditions to determine expression robustness. To infer evolutionary conservation, we used a framework of 23 species across the *Fusarium* genus including three species covered with histone methylation data. Gene expression variation is negatively correlated with gene conservation confirming that highly conserved genes show higher expression robustness. In contrast, genes marked by H3K27me3 do not show such associations. Furthermore, highly conserved genes marked by H3K27me3 encode smaller proteins, exhibit weaker codon usage bias, higher levels of hydrophobicity, show lower intrinsically disordered regions, and are enriched for functions related to regulation and membrane transport. The evolutionary age of conserved genes with H3K27me3 histone marks falls typically within the origins of the *Fusarium* genus. We show that highly conserved genes marked by H3K27me3 are more likely to be dispensable for survival during host infection. Lastly, we show that conserved genes exposed to repressive H3K27me3 marks across distantly related *Fusarium* fungi are associated with transcriptional perturbation at the microevolutionary scale. In conclusion, we show how repressive histone marks are entangled in the evolutionary fate of highly conserved genes across evolutionary timescales.

## Introduction

Highly conserved genes are ensuring housekeeping functions essential for the survival of the organism (She et al. 2009). Beyond sequence conservation, highly conserved genes are often constitutively expressed. Changes in gene expression are a major factor associated with the evolutionary trajectory of genes. In yeasts, gene expression levels are negatively correlated with the evolutionary rate of gene sequences (Pál et al. 2001; Drummond et al. 2006). The negative relationship is also termed E-R correlation (E for gene expression, R for evolutionary rate) and is broadly supported across the tree of life including bacteria (Rocha and Danchin 2004; Drummond et al. 2006), Metazoa (Krylov et al. 2003), and plants (Ingvarsson 2007). Both empirical and theoretical approaches support that the E-R correlation stems from the fact that highly expressed genes show stronger functional constraints. Such constraints include purifying selection acting against deleterious mutations affecting translation, protein folding, and maladaptive protein interactions (Zhang and Yang 2015). Additionally, there is evidence for an important role of stabilizing selection acting on expression variation over deep evolutionary timescales (Hodgins-Davis et al. 2015) and at microevolutionary scale (Kalinka et al. 2010). Despite the broad evidence that gene expression levels are strongly associated with the rate of protein evolution across species, how the association arises through genetic and epigenetic modifications over shorter evolutionary time spans remains largely unknown.

Gene expression in eukaryotes is governed largely by higher-order chromatin structure (Woodcock and Ghosh 2010). Chromatin is determined by the nucleosome, which contains a histone octamer attached to a stretch of nuclear DNA (Luger et al. 1997). Histones and DNA are constantly being modified by various proteins catalyzing enzymatic activities, such as phosphorylation, acetylation, methylation, ubiquitination, and O-GlcNAcylation. The joint effect of these post-translational modifications regulates and stabilizes gene expression during the cell cycle and development while facilitating responses to environmental stimuli (Youn 2017). For example, cytosine methylation is a covalent DNA modification associated with transcriptional responses to biotic stress (Dowen et al. 2012) and plays a key role in genomic defenses against selfish genetic elements in plants (Cui et al. 2013), mammals (Smith and Meissner 2013), and fungi (Zhou et al. 2001). Histone modifications also dynamically remodel chromatin structure to activate or repress gene expression following intrinsic and extrinsic signals (Flavahan et al. 2017). Specific histone modifications such as the methylation of H3K9 residues are frequently associated with transcriptionally silent heterochromatin, whereas acetylation and methylation of H3K4 and H3K36 residues are hallmarks of transcriptionally active euchromatin (Gates et al. 2017). The H3K27me3 modification plays a particularly important role by underpinning facultative heterochromatin allowing for responsive gene expression regulation (Trojer and Reinberg 2007).

The H3K27 residue is methylated by the protein Polycomb repressive complex 2 (PRC2; Qian et al. 2006). The canonical formation of the complex constitutes three core domains KMT6/EZH2, EED, SUZ12 which are conserved in multiple taxa such as vertebrates, insects, plants, and fungi (Ridenour et al. 2020). In *Neurospora crassa*, H3K36me modulates H3K27me3 deposition (Bicocca et al. 2018) showing that H3K27 is in cross-talk with other epigenetic marks (Du et al. 2015). H3K27 marks are often associated with transcriptional repression in gene-rich chromosomal regions (Jamieson et al. 2013), control of developmental stages in higher organisms such as plants and flies (Schwartz and Pirrotta 2007), and genome instability in fungi (Janevska et al. 2018; Möller et al. 2019). Due to its importance for both gene regulation and genome integrity, H3K27me3 is one of the best-studied post-translational marks in fungi. H3K27me3 marks show a wide range of abundance from ∼7% in the *N. crassa* genome to ∼30% in *Fusarium graminearum* (Freitag 2017). In the budding yeast *Saccharomyces cerevisiae* and fission yeast *Schizosaccharomyces pombe*, H3K27 methylation is absent due to the loss of the Polycomb complex (Freitag 2017). Experimentally induced loss of H3K27 methylation has highly variable impacts. Inactivation of the Polycomb complex (i.e., PRC2) in *Fusarium* fungi causes growth and developmental defects, as well as sterility (Freitag 2017). Interestingly, loss of the PCR2 subunit NPF described in *N. crassa* is associated with subtelomeric gene methylation (Jamieson et al. 2013) and leads to growth reductions (McNaught et al. 2020). Disruption of H3K27me3 in *Zymoseptoria tritici* is associated with chromosome destabilization (Möller et al. 2019).

Across fungi, H3K27me3 regulates the expression of rapidly evolving genes encoding virulence factors and biosynthetic pathways of specialized metabolites (Connolly et al. 2013; Jamieson et al. 2013). How facultative heterochromatin such as governed by H3K27 methylation impacts the evolution and transcriptional robustness of more conserved genes remains largely unknown. Studies have focused on deep evolutionary timescales (Fair et al. 2020) and a small number of highly conserved orthologs across taxa. However, identifying causal factors underlying among species transcriptional robustness is challenging because of environmental heterogeneity and niche differentiation (Dötsch et al. 2015). Furthermore, technical noise arising from different experimental setups renders comparisons difficult (De Jong et al. 2019) and uncertainty about orthology inference and functional divergence add additional uncertainty. In contrast, closely related fungi provide ideal models to analyze how repressive histone marks underpin the robustness of transcription and sequence conservation across species. The ability to assess transcriptional robustness in identical experimental settings in species sharing collinear genomes, similar habitats, and life cycles is a key advantage to make robust inferences about causal factors.

Fungi of the genus *Fusarium* are important pathogens of crops causing a wide range of diseases (Summerell 2019). Originally described as a single species (O’Donnell et al. 2004), the *F.graminearum* species complex (FGSC) comprises 16 recognized species (O’Donnell et al. 2008). All species can cause Fusarium head blight in cereals and hence have strongly overlapping host ranges (van der Lee et al. 2015). FGSC are all closely related (Walkowiak et al. 2016; Tralamazza et al. 2019) but encode a vast and variable repertoire of specialized metabolites (Tralamazza et al. 2019). The genome of *F. graminearum* has one of the most strongly affected gene bodies in terms of repressive H3K27me3 marks among fungi (46% of genes; Freitag 2017) making FGSC an ideal model to analyze epigenetic control of gene transcription.

To investigate the impact of repressive histone marks on gene transcription robustness and protein conservation across species, we performed genome-wide transcription analyses of five FGSC members including the reference genome strain of *F. graminearum* (PH-1; Cuomo et al. 2007). We analyzed transcriptional responsiveness across two environmental conditions including the infection of the wheat host and a nutrient-rich growth medium. To infer evolutionary conservation of coding sequences, we used a comparative genomics framework of 23 species across the genus of *Fusarium* spanning approximately 100 My of evolution (Summerell et al. 2010; O’Donnell et al. 2013). We integrated histone methylation and transcriptomics data from three *Fusarium* species across the phylogenetic breadth of the genus. We found that gene transcription variation is negatively correlated with gene conservation across the genus confirming that highly conserved genes show higher gene transcription robustness. Genes consistently silenced through H3K27me3 histone marks across *Fusarium* showed higher gene transcription variation among species. Marked genes encoded for on average smaller proteins, showed lower GC content, codon usage bias and were enriched for functions related to regulation and membrane transport. Estimates of the evolutionary age of conserved genes showed that genes with H3K27me3 histone marks are of much more recent origin than unaffected genes. Lastly, we show that highly conserved genes marked by H3K27me3 are more likely to be dispensable.

## Results

### Robustness of Transcriptomic Responses among Closely Related Species

**Fig. 1.**
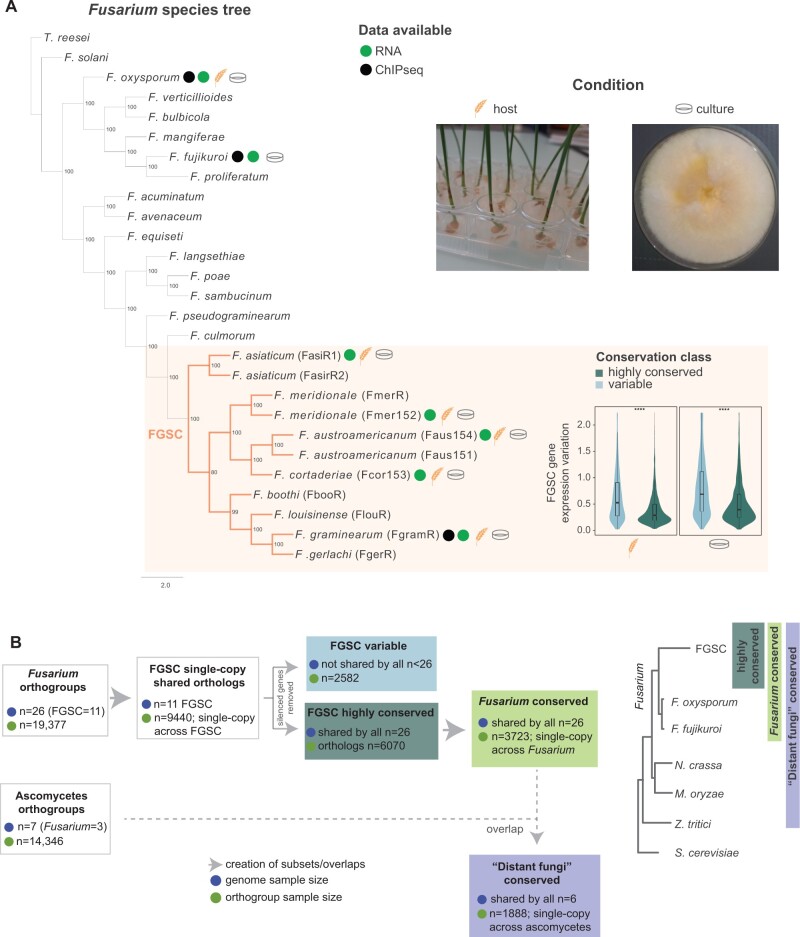
The *Fusarium* genus and FGSC as a histone methylation model system. (*A*) The phylogenomic tree of *Fusarium* species was inferred from a coalescence-based analysis of 4,192 single-copy orthologs (Tralamazza et al. 2019). Values indicate support from 100 bootstrap replicates. *Trichoderma reseei* was used as an outgroup. Colored circles indicate data type utilized in the current study. Host and culture medium describe the two environmental conditions. The violin plot refers to gene transcription variation of highly conserved versus variable genes in FGSC (Wilcoxon test, *P*-value < 0.0001). (*B*) Outline of the data sets used in the study. To analyze the association of transcription and histone marks, single-copy orthologs shared among FGSC species (*n* = 9,440) were selected. To infer transcriptional robustness and gene dispensability, we removed silenced genes from the data set and defined conservation categories (highly conserved vs. variable data sets). To infer transcriptional perturbation across *Fusarium*, we subset for single-copy genes across the *Fusarium* genus (“*Fusarium* conserved”). We used single-copy orthologs of model ascomycetes and matched this with the “*Fusarium* conserved” data set to analyze the conservation of marks at the deepest phylogenetic level (“distant fungi” data set).

We performed transcriptomic profiling on closely related fungi belonging to the *F.graminearum* species complex (FGSC; fig. 1). The group of fungi includes crop pathogens infecting mainly wheat. The highly overlapping habitat and lifestyle together with the recent history of speciation make FGSC highly suitable to assess the impact of repressive histone modifications on gene conservation and transcription robustness. We first assessed the degree of protein sequence conservation within the complex using orthology and found that for a total of 14,346 detected orthogroups, 9,440 orthogroups were composed of single-copy genes shared among all analyzed FGSC members (supplementary table S3 and fig. S1*A*, Supplementary Material online). Then, we expanded the orthology analysis to 23 species covering the entire genus *Fusarium*. From the single-copy genes shared among all analyzed FGSC members, we find 6,243 (66.1%) orthologs to be conserved genes among all 26 *Fusarium* (fig. 1*B*). To assess gene transcription robustness under standardized environmental conditions, we generated RNA-seq data for five FGSC members during infection of wheat and growth on nutrient-rich medium (fig. 1*A*). The transcriptomic data showed high reproducibility (supplementary fig. S1*B*, Supplementary Material online). We detected reliable transcription in ∼92% of all single-copy genes among FGSC members (*n =* 8,666/9,440). Most genes (87.7%) were transcribed in all five species during wheat infection and on growth medium (82.8%; supplementary table S4, Supplementary Material online). Highly conserved genes showed higher gene transcription robustness compared with variable genes (fig. 1*A*).

### Chromatin Landscape of the *F. graminearum* Genome and Transcription Robustness

**Fig. 2.**
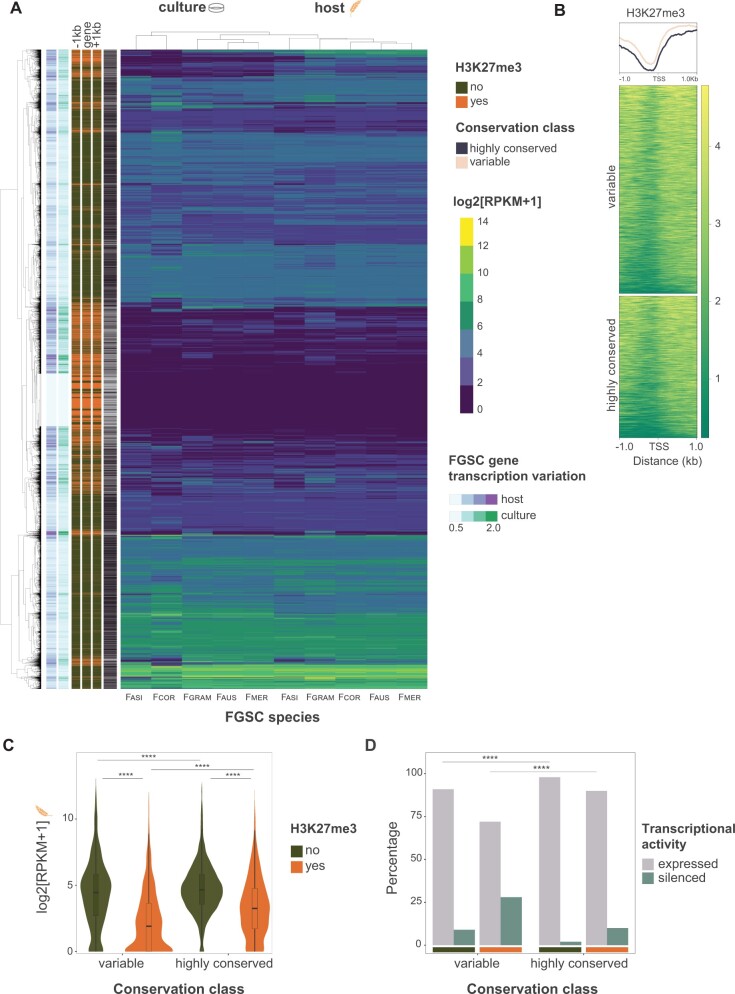
Genome-wide transcription and transcription variation profile of the FGSC based on single-copy orthologs, *n* = 9,440. (*A*) Transcriptome analysis of the FGSC based on hierarchical clustering (complete linkage method). Host and culture medium describe the two environmental conditions. Green and orange colors indicate the presence or absence of H3K27me3 in the gene body, as well as 1 kb upstream and downstream of genes based on Homer peak calling. (*B*) Signal profiles in the 2 kb around TSSs in *Fusarium graminearum* genes marked by H3K27me3. (*C*) Mean transcription of conserved and variable genes during host infection in the FGSC. Wilcoxon test adjusted *P*-value < 0.0001. (*D*) Percentage of silenced genes according to H3K27me3 coverage and conservation category during host infection. Two-proportion *z*-test, *P* < 0.0001. Fgram, *F. graminearum*; Faus, *F. austroamericanum*; Fcor, *F. cortaderiae*; Fmer, *F. meridionale*; Fasi, *F. asiaticum*.

Following the association of gene conservation and transcriptional robustness within FGSC, we analyzed links between chromatin modification marks and transcription profiles of individual genes (fig. 2). For this, we assessed the occupancy of histone modifications in gene body regions of the reference genome *F. graminearum*. We first investigated H3K27me3 marks of *F. graminearum* growing in high nitrogen and low nitrogen medium (Connolly et al. 2013). We found a high correlation gene body methylation between conditions (*r* = 0.99, Spearman correlation, *P* < 2.2e−16; supplementary fig. S2*A*, Supplementary Material online). A small set of 62 genes showed incongruent H3K27me3 methylation between conditions with the low nitrogen medium showing higher methylated gene counts (supplementary fig. S2*B* and table S4, Supplementary Material online). We pursued further analyses exclusively with the data set from low nitrogen media. The extent of gene body H3K27me3 marks is strongly bimodal with either no or nearly no marks contrasted with dense gene body coverage (supplementary fig. S2*B* and C, Supplementary Material online). Hence, we categorized genes into marked (*n* = 2563; with >50% gene body coverage) or unmarked by H3K27me3 (*n* = 6877; <50% coverage), respectively (supplementary fig. S2*D*, Supplementary Material online). Variable genes were enriched in H3K27me3 marks compared with highly conserved genes (*P* < 0.0001; supplementary fig. S2*D*, Supplementary Material online). As expected, the gene body methylation profile shows a strong association with the upstream region and a drop near the transcription start site (TSS; fig. 2*B*). Transcriptional profiling of genes within the species complex revealed strong genome-wide associations between gene transcription levels, transcription robustness, protein sequence conservation, and H3K27me3 gene marks (fig. 2*A*). We found that conserved, unmarked genes were the most highly expressed genes (median of 24.2 RPKM; fig. 2*C*). Furthermore, conserved marked genes were less repressed (median 8.53 RPKM, with 10% showing no transcription) than variable marked genes (median of 2.76 RPKM, with 28% showing no transcription; fig. 2*C* and *D*; *P* < 0.0001).

**Fig. 3.**
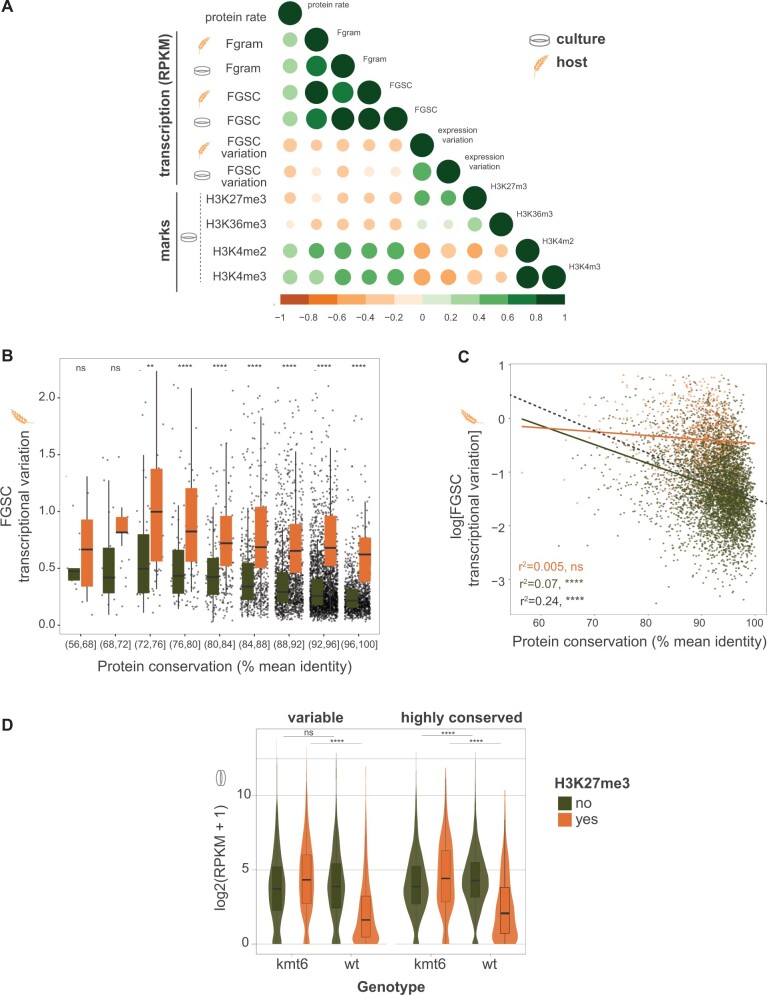
Analyses of gene transcription and gene body histone methylation marks among FGSC. (*A*) Correlation plot between marks and conditions. Spearman's rank tests were performed for paired samples. Circles indicate *P*-values < 0.0001. Circle size and color indicate degrees of correlation. Culture and host describe growth conditions. Fgram, *F. graminearum*. FGSC—mean value of the *F. graminearum* species complex. FGSC variation—coefficient of variation based on each species-specific RPKM values. (*B*) Distribution of transcriptional variation of highly conserved genes (*n* = 6,070) during host infection based on protein sequence conservation. (*C*) Correlation between gene transcription variation and protein sequence conservation. Gray *r*^2^ value refers to multiple regression model for gene transcriptional variation as a function of protein conservation and H3K27me3 marks. The dashed gray line refers to the linear regression slope of the full data set of highly conserved genes. Colored lines and respective *r*^2^ values refer to the linear regression for H3K27me3 marked genes (orange, *n* = 810) and unmarked genes (green, *n* = 5,260). (*D*) Gene transcription analysis of the *F. graminearum* wild-type strain (wt) and mutant lacking the methyltransferase gene *kmt6* (*Δkmt6*) assessed under culture conditions (Connolly et al. 2013). For the violin and boxplots, Wilcoxon two-sided paired and unpaired tests were performed, respectively. ns: *P* > 0.05, *P ≤ 0.05, **P ≤ 0.01, ***P ≤ 0.001, ****P ≤ 0.0001.

To identify general patterns how H3K27me3 marks impact sequence conservation and gene transcription robustness, we first removed genes silenced in all of the FGSC (788 out of 9,440) and both conditions tested (i.e., culture and on host). Then, we then expanded the analyses to the 6,072 highly conserved of the *Fusarium* genus (comprising 810 H3K27me3 marked and 5,260 unmarked genes). The data set included a further 2,582 variable genes (comprising 1,188 H3K27me3 marked and 1,394 unmarked genes). Highly conserved genes of *F. graminearum* share a 92.2% mean amino acid identity with orthologs within the genus (data not shown). We find support for a general E-R correlation (fig. 3*A*). Protein sequence conservation shows significant positive correlations with gene transcription levels within species (*F. graminearum* on host; *r* = 0.37; *P* < 0.0001) and between species (among FGSC members on host; *r* = 0.39; *P* < 0.0001). We found that gene transcription variation was negatively correlated (*P*-value < 0.0001) with protein sequence conservation (*r* = −0.32 and −0.25 of FGSC members on host and in culture, respectively; fig. 3*A*). We found a positive correlation with histone H3K27me3 marks and gene transcription variation (i.e., lower robustness; *r* = 0.41) during infection and culture condition (*P*-value < 0.0001; fig. 3*A*). As expected, euchromatin marks H3K4m2/3 showed a positive correlation with gene transcription (H3K4m2/3 among FGSC members on host; *r* = 0.44) and a negative correlation with transcription variation (H3K4m2/3 on host; *r* = −0.46). We then tested for the correlation of gene transcription variation with H3K27me3 marks controlling for the presence H3K4m2 marks to estimate independent effects of H3K27me3 (supplementary fig. S3*A*, Supplementary Material online). The correlation of transcription variation on the host with H3K27me3 marks remains positive (*r* = 0.28), yet additional confounding factors likely remain unaccounted for.

To further investigate the association of H3K27me3 marks and transcription, we analyzed gene transcription and transcriptional robustness among members of the FGSC. Highly conserved genes (i.e., shared among the 26 species) marked by H3K27me3 are on average more divergent than unmarked genes (89.3% and 92.7% mean protein identity, respectively: supplementary fig. S3*B*, Supplementary Material online). Gene transcription is positively associated with protein sequence conservation both on the host and under culture conditions (supplementary fig. S3*C*, Supplementary Material online). Highly conserved genes with H3K27me3 marks show broad transcriptional repression (fig. 3*B*), yet a general E-R association is maintained (linear regression *r*^2^ = 0.13, *P* < 0.0001). Next, we analyzed gene transcription variation among members of the FGSC. Higher protein conservation was associated with reduced gene transcription variation (i.e., higher robustness) both on the host and in culture condition (fig. 3*B*; supplementary fig. S3*D*, Supplementary Material online). Such a general association is consistent with the idea that highly conserved genes are under stabilizing selection to retain transcriptional activity independent of the genetic environment (i.e., the species identity). Next, we examined the impact of H3K27me3 on robustness and found that marked genes showed significantly higher levels of gene transcription variation (fig. 3*B*, *P* < 0.0001). Remarkably, the association between gene transcription variation and sequence conservation found for H3K27me3 unmarked genes (fig. 3*C*; linear regression *r*^2^ = 0.07, *P* < 0.0001) is substantially reduced for marked genes (fig. 3*C*; *r*^2^ = 0.005, *P* = 0.03). We added presence of H3K27me3 marks as factor to a model explaining transcriptional variation from gene conservation. We find that H3K27me3 explained a significant fraction of transcriptional variation (24% or *r*^2^ = 0.24, *P* < 0.0001; fig. 3*C*). Taken together, our analyses show that H3K27me3 is associated with a loss of correlation between transcriptional robustness and protein sequence conservation.

To investigate if the loss of transcription is a consequence of H3K27me3 marks, we analyzed RNA-seq and chromatin immunoprecipitation sequencing (ChIP-seq) data set of a *F. graminearum kmt6* mutant strain (Connolly et al. 2013). The mutant lacks the methyltransferase enzyme KMT6 responsible for H3K27me3 marks in fungi (Freitag 2017). We found that conserved genes that lost H3K27me3 marks in the *kmt6* mutant have transcription levels similar to conserved genes that are unmarked in the wild-type background (fig. 3*D*, *P* > 0.05). Less conserved (i.e., variable) genes showed similar patterns of derepression in the *kmt6* mutant background but overall lower levels of transcription (fig. 3*D*, *P* < 0.0001).

### Feature Enrichment of H3K27me3 Marked Genes

**Fig. 4.**
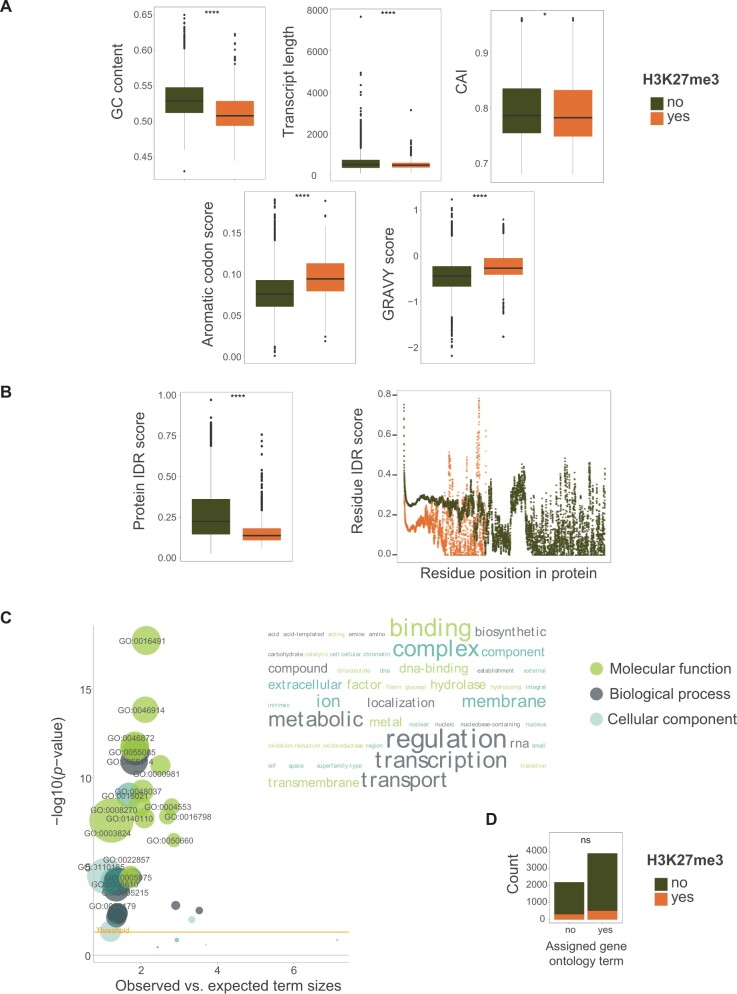
Features of H3K27me3 marked (*n* = 810) and unmarked genes (*n* = 5,260). (*A*) Analyses of codon and amino acid features of highly conserved genes encoded in the *Fusarium graminearum* genome. Green and orange colors indicate the presence or absence of H3K27me3 marks in the gene body. CAI refers to the codon adaptation index, GRAVY scores amino acid hydrophobicity. (*B*) Boxplot of IDR mean score per protein sequence. The scatter plot shows IDR mean scores per amino acid position. A Wilcoxon two-sided test was performed. (*C*) GO term enrichment analysis of highly conserved genes marked by H3K27me3. The word size represents the enriched GO term size. The threshold line indicates *P* < 0.0001 (Fisher test). (*D*) Proportion of marked and unmarked genes with assigned GO terms. Two-proportion *z*-test. ns: *P* > 0.05, *P ≤ 0.05, **P ≤ 0.01, ***P ≤ 0.001, ****P ≤ 0.0001.

We investigated protein functions encoded by highly conserved genes (*n* = 6070). We found that H3K27me3 marked genes have shorter transcripts than unmarked genes (median 452 vs. 486 bp; *P* < 0.0001) and have a lower GC content (51% vs. 53%; *P* < 0.0001; fig. 4*A*). We found that highly conserved genes covered by H3K27me3 marks have significantly less codon usage bias than unmarked genes (codon adaptation usage [CAI] median of 0.778 vs. 0.785; *P* < 0.05). The encoded proteins differ also in amino acid composition with an aromatic codon score median of 0.093 and 0.075 (*P* < 0.0001) for marked and unmarked genes, respectively. We evaluated thermodynamic properties related to protein interaction and folding. Marked genes have higher amino acid hydrophobicity compared with unmarked genes (median of −0.260 vs. −0.430, *P* < 0.0001; fig. 4*A*). Conserved genes with H3K27me3 marks and unmarked genes also differ significantly at the level of intrinsically disordered regions (IDRs) with median scores of 0.137 and 0.224, respectively (*P* < 0.0001; fig. 4*B*). Gene ontology (GO) terms were assigned overall to 3,878 out of 6,070 coding sequences with no significant difference between marked and unmarked genes (*P* > 0.05). Conserved genes with H3K27me3 marks are enriched for functions related to oxidation-reduction, transmembrane functions, and transcriptional regulation (*P*-value < 0.0001; fig. 4*B*, supplementary fig. S4*A*, Supplementary Material online). Highly conserved unmarked genes are enriched for housekeeping functions such as cellular metabolic and macromolecular processes (supplementary fig. S4*B*, Supplementary Material online).

### Evolutionary Origins of Highly Conserved Genes

**Fig. 5.**
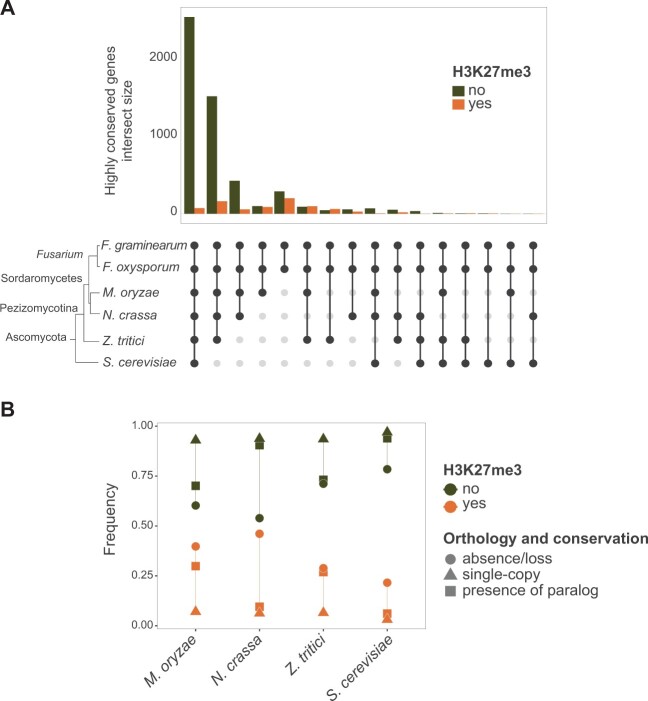
Phylogenetic analyses of highly conserved genes. (*A*) Highly conserved genes in FGSC with orthologs among ascomycetes (*n* = 6,070; see fig. 1*B*). (*B*) Frequency of gene absence/loss, single-copy orthologs, and presence of a paralog for H3K27me3 marked and unmarked genes across distant ascomycete. Orange and green colors indicate the presence and absence of H3K27me3 in the gene body, respectively.

To investigate the evolutionary origins of highly conserved genes in FGSC (*n* = 6,070), we expanded the orthology analyses to a set of model ascomycetes including *N. crassa*, *Magnaportheoryzae*, and *Z. tritici* (fig. 5*A*). Orthology analyses showed that the majority of the unmarked genes are shared among ascomycetes (fig. 5*A*; supplementary table S5, Supplementary Material online). In contrast, most H3K27me3 marked genes are not found outside of the *Fusarium* genus (fig. 5*B*). Interestingly, orthologs between *F. graminearum* H3K27me3 marked genes and ascomycete fungi are enriched for having paralogs among ascomycete fungi compared with unmarked genes (supplementary fig. S5*A*, Supplementary Material online). The creation of gene duplicates can trigger genomic defense mechanisms such as repeat-induced point (RIP) mutations in fungi (Galagan and Selker 2004). We analyzed evidence for RIP-like mutations in the gene sets of *F. graminearum* but found only a small portion of the entire genome to be affected (<1.1%). We found no difference between the extent of RIP-like mutations in H3K27me3 marked versus unmarked genes (supplementary fig. S5*B*, Supplementary Material online).

### Evolutionary Conservation of H3K27me3 Marks among *Fusarium* Fungi

**Fig. 6.**
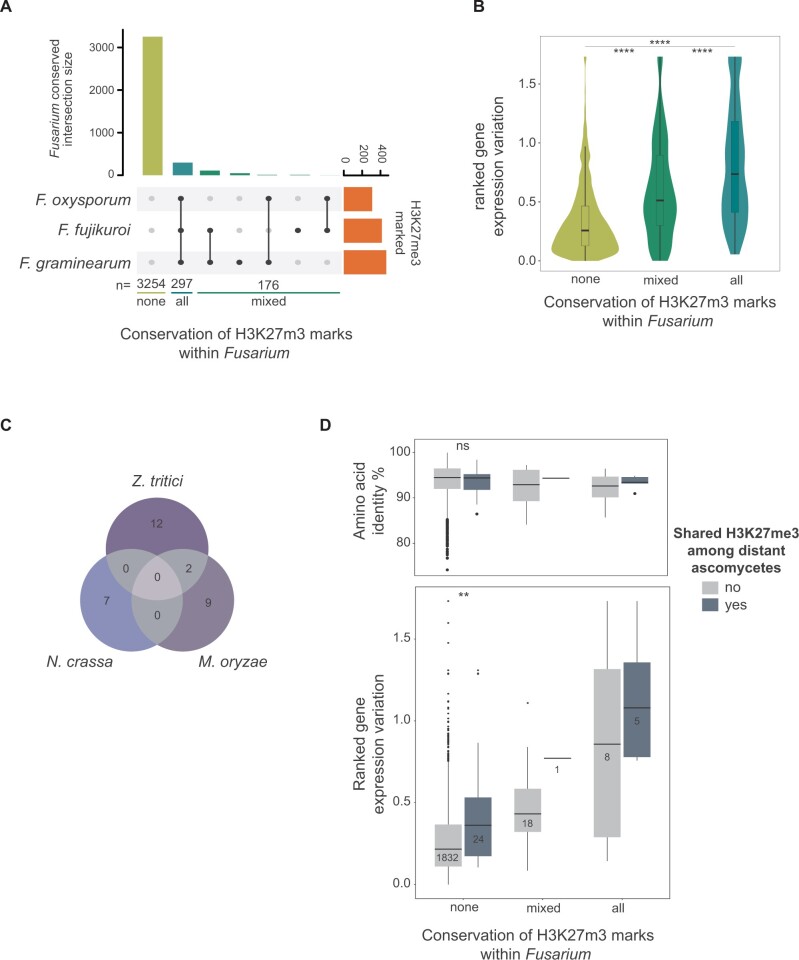
Conservation of H3K27me3 gene body occupancy in genes conserved in *Fusarium* (*n* = 3,727). (*A*) Co-occurrence of H3K27me3 marks in *Fusarium graminearum*, *F. oxysporum*, and *F. fujikuroi.* (*B*) Ranked relative gene transcription based on shared H3K27me3 marks of *Fusarium* species under culture growth conditions. (*C*) Single-copy orthologs shared between the distant ascomycetes *Neurospora crassa*, *Magnaporthe oryzae*, and *Zymoseptoria tritici* (“distant fungi” data set, *n* = 1,880; see also fig. 1*B*). Only orthologs with H3K27me3 gene body occupancy are shown (*n* = 30). (*D*) Comparison of genes conserved in *Fusarium* and among distant ascomycetes (i.e., *Fusarium* and “distant fungi” data sets). Mean amino acid identity and ranked gene transcription variation for each group. Wilcoxon tests with adjusted *P*-values (Holm method). ns: *P* > 0.05, *P ≤ 0.05, **P ≤ 0.01, ***P ≤ 0.001, ****P ≤ 0.0001. Due to small sample size, no tests were performed for the *all* and *mixed* categories.

To understand the evolutionary longevity of epigenetic effects on gene transcription robustness, we analyzed H3K27me3 marks across the genomes of distantly related *Fusarium* species spanning ∼100 My of divergence. We examined 3,727 highly conserved single-copy orthologs shared by *F. graminearum*, *F. oxysporum*, and *F. fujikuroi* (fig. 1*A*; *Fusarium conserved* data set). Based on gene age calibrations for *F. oxysporum*, we find that unmarked conserved genes in *Fusarium* have a median age of ∼439 My. In contrast, the median evolutionary age of H3K27me3 marked genes is ∼394 My (supplementary fig. S6*A*, Supplementary Material online) matching the upper range of the estimated age of the *Fusarium* genus (Summerell et al. 2010). As expected, the large majority of the genes conserved in *Fusarium* are not covered by histone H3K27me3 marks (*n* = 3,254; fig. 6*A*). Among genes marked by H3K27me3, 62.7% (*n* = 297/473, *all* category in fig. 6*B*) shared the mark among the three *Fusarium* species and 37.2% showed inconsistent marks (*n* = 176/473; fig. 6*A*; *mixed* category in fig. 6*B*). This suggests that H3K27me3 is often retained over significant evolutionary timescales. Next, we asked if H3K27me3 mark conservation in *Fusarium* is correlated with transcriptional robustness. Using ranked gene transcription, we compared gene transcription variation and ChIP-seq coverage among *F. oxysporum*, *F. fujikuroi*, and *F. graminearum*, in culture condition. Genes with H3K27me3 marks shared among all *Fusarium* show higher transcription variation than other genes (*all* category in fig. 6*B*). Conversely, unmarked genes showed the lowest transcriptional variation (i.e., highest robustness; *none* category in fig. 6*B*). Interestingly, genes without conserved H3K27me3 marks (*mixed* category) show intermediate transcriptional robustness compared with the other categories (fig. 6*B*). Overall, transcriptional robustness associated with H3K27me3 marks is found consistently across the *Fusarium* genus.

### H3K27me3 Marks of Highly Conserved Genes in Ascomycetes

To investigate repressive histone marks and protein conservation beyond the origins of the *Fusarium* genus, we analyzed single-copy orthologs shared between *Fusarium* and the distantly related model ascomycetes *N. crassa*, *M. oryzae*, and *Z. tritici* (*n* = 1888, “distant fungi conserved” data set, fig. 1*B*). *Saccharomycescerevisiae* was not included due to the lack of H3K27me3 (Freitag 2017). We found no instance of an ortholog sharing H3K27me3 among all distant fungi (fig. 6*C*). From the 30 orthologs with H3K27me3 gene body occupancy in the “distant fungi conserved” data set, we found five orthologs that shared the H3K27me3 mark also with *Fusarium* species (see “all” category in fig. 6*D*). We found that the orthologs conserved between *Fusarium* and the other ascomycetes showed similar variation in transcriptional responses across environments for *Fusarium* species (fig. 6*D*). Interestingly, genes lacking H3K27me3 marks in *Fusarium* but share marks among distant ascomycetes show higher variation in transcription in *Fusarium* species (*P* < 0.01; fig. 6*D*). The analyses suggest similar trends for genes with H3K27me3 marks in *Fusarium*; however, sample size was too low for meaningful significance testing. Hence, despite similar degrees of protein conservation (fig. 6*D*), the presence of H3K27me3 marks in distant ascomycetes is associated with higher transcriptional variation (i.e., lower robustness) within the *Fusarium* genus.

### Links between H3K27me3 Marks and Gene Dispensability

**Fig. 7.**
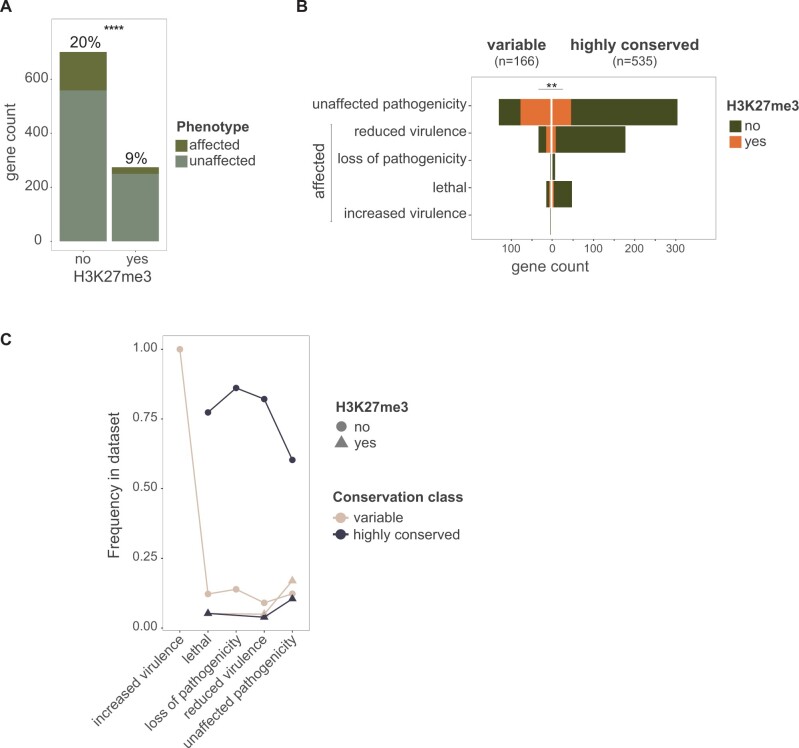
Phenotypic effect in gene deleted mutants of *Fusarium graminearum* during host infection based on the PHI-base database (*n* = 701 genes). (*A*) Distribution of affected and unaffected phenotypes during infection (i.e., increased virulence, lethality, loss of pathogenicity, reduced virulence). (*B*) Phenotypic effects of mutants as a function of the conservation category and the presence or absence of H3K27me3 in the gene body. (*C*) Frequency of each phenotype category. Two-proportion *z*-test. ns: *P* > 0.05, *P ≤ 0.05, **P ≤ 0.01, ***P ≤ 0.001, ****P ≤ 0.0001.

To investigate the association of H3K27me3 marks and gene dispensability, we focused on databases reporting phenotypic effects of deletion mutant lines for *F. graminearum* genes. A total of 1,066 genes have reports of mutant phenotypes in the pathogen–host interaction (PHI)-base (supplementary table S2, Supplementary Material online). Screened phenotypes include increased/reduced virulence, loss of pathogenicity, and lethality. We retained 873 mutants by overlapping the data set with the genes comprised in our single-copy shared orthologs data set (*n* = 9,040; fig. 1*B*). A total of 83 genes have reports for multiple phenotypes (e.g., for different hosts or host tissue). For these genes, we randomly selected a single phenotype and used the mean distribution (50 repetitions; supplementary fig. S7*A*, Supplementary Material online). Among the mutant set, H3K27me3 marked genes have overall a lower proportion of genes with an overt phenotypic effect during host infection compared with unmarked genes (*P* < 0.0001; fig. 7*A*). Highly conserved genes showed a higher proportion of genes with an overt phenotypic effect (232/533, ratio of 0.43) compared with variable genes (40/166, ratio of 0.24; two-proportion *z*-test, *P* < 0.0001). Analyzing the association with the degree of gene conservation within the FGSC, we find that highly conserved H3K27me3 marked genes (*n* = 56) had a lower proportion of genes with an overt phenotypic effect compared with variable H3K27me3 marked genes (*n* = 86; *P* < 0.01; fig. 7*B*). The sample size per mutant phenotype category was too low to investigate associations with H3K27me3 or conservation status. The available data suggest that H3K27me3 marked genes show proportionally lower overt phenotypic effects in lethality, loss of pathogenicity, and reduced virulence in marked genes independent of the conservation category (fig. 7*C*). Finally, we analyzed a data set of 101 *F. oxysporum f. sp. lycopersici* mutants with reduced pathogenicity compared with the wild type (Michielse et al. 2009). We found that only 7% (7/101) of the genes were marked with H3K27me3 compared with the genome-wide proportion of H3K27me3 marked genes of 30% (7,633/20,925; supplementary fig. S7*B*, Supplementary Material online). Hence, H3K27me3 marks are tightly linked to gene dispensability in the *Fusarium* genus and the association is tighter in highly conserved genes compared with variable genes (fig. 7*A*).

## Discussion

We analyzed a major group of plant pathogenic fungi to understand factors associated with gene transcription robustness over deep evolutionary timescales. We performed transcriptome-wide analyses to assess transcription patterns using matching conditions across species. Integrating histone marks data across the genus and additional ascomycetes, we identified the likely impact of methylation patterns on transcriptional robustness, protein conservation, and gene dispensability.

### Repressive Histone Marks Predict Gene Transcription and Dispensability

Highly conserved genes tend to be constitutively expressed to maintain basic cellular functions (She et al. 2009). We show that highly conserved genes marked by H3K27me3 deviate from these archetypical housekeeping properties. Compared with unmarked genes, marked genes exhibit shorter transcripts, lower GC content, and weaker codon usage bias, which are features associated with fast-evolving genes (Lipman et al. 2002; Jamieson et al. 2013; Zhang and Yang 2015). Functions encoded by marked genes were also enriched for transcriptional regulation and binding functions. This is consistent with findings in the *Arabidopsis* genus with an enrichment for similar functions (Ha et al. 2011). The higher levels of aromatic codons and hydrophobicity of proteins encoded by marked genes suggest weakened selection against misfolding and aggregation (Bastolla et al. 2004). In contrast, highly conserved genes are typically under strong purifying selection to encode key functions in molecular and cellular processes. Similarly, highly expressed genes are under strong selection for transcription robustness to reduce translational errors, protein misfolding and to avoid promiscuous protein–protein interactions (Zhang and Yang 2015; Agozzino and Dill 2018). Destabilized proteins can cause protein–protein aggregation with deleterious consequences for cellular functions including membrane integrity (Stefani and Dobson 2003). IDRs are protein regions showing folding heterogeneity providing potential plasticity for protein interactions with other molecules. Proteins with a high IDR tend to have important roles in regulation and essential cellular processes (Wright and Dyson 2015). Protein regions with IDR can achieve high specificity with low affinity, prevent protein aggregation, and provide resistance to non-native condition (e.g., resistance to unfolding due to environmental perturbation; Liu and Huang 2014). Interestingly, H3K27me3 marked genes are depleted for IDR regions compared with unmarked genes. Our analyses show also that highly conserved genes marked by H3K27me3 encode distinct functions compared with similarly conserved genes without heterochromatin marks. In conjunction, H3K27me3 unmarked genes tend to share characteristics associated with transcriptional robustness and resistance to perturbation compared with marked genes.

We find that mutant lines for highly conserved genes are more likely to generate an overt phenotypic defect during host infection compared with less conserved genes consistent strong selection underlying sequence conservation. In the same analyses, H3K27me3 marked genes were less likely to generate an overt phenotypic defect compared with unmarked genes. A lack of apparent phenotypic consequences could stem from genetic redundancy. However, our analysis focused on single-copy orthologs making gene duplications an unlikely explanation (i.e., by creating redundancy). Alternatively, genes marked by H3K27me3 could have recently lost essentiality either through changes in the environmental interactions of the organism or mutations in the genetic background creating redundancy (Bergmiller et al. 2012). Interestingly, essential genes marked by H3K27me3 can become dispensable through epistatic interactions, monogenic suppressors, or similar mechanisms (Li et al. 2019; Rousset et al. 2021). Genomic regions with repressive histone marks may coincide with higher densities of repetitive elements. In some fungi, such regions are targeted by genomic defense mechanisms including RIP (Galagan and Selker 2004) causing loss-of-function mutations (Möller et al. 2021). We found no evidence for higher rates of RIP-like mutations in marked versus unmarked genes. Hence, gene dispensability most likely evolved through other mechanisms.

### H3K27me3 Perturbs Transcriptional Robustness of Highly Expressed Genes

Highly conserved genes in the *Fusarium* genus show significant positive associations between gene transcription and degree of conservation. Hence, our results confirm the E-R correlation observed broadly among eukaryotes (Zhang and Yang 2015). Beyond levels of transcription, we found that transcription robustness is strongly associated with the degree of protein conservation independent of the environment (i.e., growth condition). The association likely reflects selection acting on regulatory elements to retain transcription homeostasis under external and internal perturbations (Kitano 2004). H3K27me3 plays a significant role in gene transcription robustness among closely related species. Less conserved genes covered by H3K27me3 are more transcriptionally silenced confirming well-documented patterns in fungi (Freitag 2017) and other eukaryotes (Zhang and Yang 2015). We find that highly conserved genes covered by H3K27me3 tend to suffer from partial transcriptional repression and rarely complete silencing. Hence, genes retained transcriptional proficiency despite the repressive nature of the histone modifications. Robustness typically evolves in order to tolerate environmental perturbations and facilitates the evolvability of complex systems (Kitano 2004; Liu et al. 2020). How gene transcription evolves through the combined effects of mutations, drift, and selection acting on regulatory variation has received considerable attention. A key issue is to determine the relative importance of the different evolutionary forces. In a well-controlled modeling study of gene transcription variation across organisms, the “House-of-Cards” model received the strongest support (Hodgins-Davis et al. 2015). The model (Kingman 1978; Turelli 1984) stipulates infrequent mutations of large effect tied to strong stabilizing selection acting on regulatory variation. Quantifying effect sizes of mutations linked to changes in the chromatin landscape (e.g., H3K27me3) could be used to expand investigations of expression quantitative trait loci. Ultimately, this would expand our understanding how selection drives the evolution gene regulation.

We found that highly conserved genes with repressive histone marks showed reduced transcriptional robustness compared with unmarked genes. Genes with repressive histone marks show no clear association between protein conservation and transcriptional robustness compared with unmarked genes. The lack of robustness could be a consequence of polymorphism in H3K27me3 marks within the species complex. However, we found that gene body H3K27me3 marks of highly conserved genes are largely conserved across the *Fusarium* genus. Associations of histone modifications and gene transcription across species were found previously in primates (Zhou et al. 2014). However, much more extensive work was done on cytosine DNA methylation. Interestingly, conservation of gene body methylation in coding sequences is positively correlated with both gene transcription levels and slower protein sequence evolution, but negatively correlated with gene transcription robustness (Chuang and Chiang 2014; Seymour and Gaut 2020). Our analyses also suggest that the reduced transcriptional robustness of H3K27me3 marked genes within the species complex is shared among *Fusarium* species, spanning a significantly longer evolutionary time scale. Robust comparisons across additional ascomycetes are challenging due to limitations in data sets and heterogeneity in environmental conditions.

Our study establishes evolutionary links between transcription robustness and protein evolution mediated by repressive histone modification. However, cross-talk between different epigenetic markers needs to be further examined to establish direct causal relationships. We show that highly conserved genes marked by H3K27me3 are transcriptionally active despite the repressive nature of the mark but suffer from perturbed transcription robustness compared with unmarked genes. Highly conserved marked genes show enrichment in environmental stress-related functions, carry hallmarks of fast-evolving genes, result in low phenotypic response during host infection and, hence, do not follow the housekeeping gene archetype. We show that H3K27me3 marks can blur the general E-R correlation of sequence conservation and transcription levels. Hence, histone modifications provide a key association between protein evolvability and gene essentiality.

## Materials and Methods

### Infection Assay

RNA-seq analyses were performed on fungal mycelium grown in culture and in planta for five species of the FGSC (*F. graminearum*, *F. meridionale*, *F. cortaderiae*, *F. asiaticum*, and *F. austroamericanum*; fig. 1*A*). Each species was grown in a Petri dish containing V8 agar medium for 4 days at 25 °C. Following the culturing, the mycelium was transferred to a 100 ml mung bean liquid medium and agitated at 170 rpm for 5 days at 25 °C (Zhang et al. 2012). Next, the medium was filtered, and spores counted using a hemocytometer. A 10^6^ per ml spore solution was prepared for the following infection procedure. For in vitro assays, cultures were inoculated from spore solutions in yeast sucrose agar medium for 72 h at 25 °C until RNA extraction. For in planta assays, wheat coleoptiles with intermediate susceptibility to Fusarium head blight (cultivar CH Combin, harvest 2018–2019) were infected with each species individually according to Zhang et al. (2012). Briefly, wheat seeds were soaked in sterilized water for germination in a culture chamber at 25 °C with a 12 h white light cycle and 93% humidity. After 3 days of germination, the tip of the coleoptile was cut and 10 µl of the spore solution was used for inoculation. Coleoptiles were collected 72 h after inoculation (∼0.4 mm lesion size) and processed for RNA extraction. All in planta assays were performed in triplicates and each replicate was composed of a pool of 25 infected seedlings to obtain sufficient material and homogenize infection conditions.

### RNA Extraction and Sequencing

RNA extraction was performed using the NucleoSpin RNA Plant and Fungi kit (Macherey‐Nagel GmbH & Co. KG, Düren, Germany) according to the manufacture’s recommendation. The RNA quality was assessed using a Qubit (Thermo Fisher Scientific, Waltham, USA) and an Agilent Bioanalyzer 2100 (Agilent Technologies, CA, USA). The NEB Next Ultra RNA Library Prep (NEB, CA, USA) kit based on the polyA method was used for RNA library preparation. Samples were sequenced on a NovaSeq 6000 system (Illumina Inc., CA, USA) and 150 bp paired-end reads were generated. Library preparation and sequencing were performed by Novogene Co., Ltd, Beijing, China.

### RNA-Seq Read Alignment and Transcript Quantification

Illumina raw reads were trimmed and filtered for adapter contamination using Trimmomatic v. 0.32 (parameters: ILLUMINACLIP: Trueseq3_PE.fa:2:30:10 LEADING:3 TRAILING:3 SLIDINGWINDOW:4:15 MINLEN:36; Bolger et al. 2014). Filtered reads were aligned using Hisat2 v. 2.0.4 with default parameters (Kim et al. 2019) to the *F.graminearum* PH-1 reference genome (King et al. 2017). Mapped transcripts were quantified using HTSeq-count (Anders et al. 2015). Read counts were normalized based on the trimmed mean of *M*-values (TMM) method using the calcNormFactors option. To account for gene length, we calculated reads per kilobase per million mapped reads (RPKM) values using the R package edgeR (Robinson et al. 2010). Gene transcription robustness (i.e., gene transcription variation) among the five species of the FGSC was calculated using the coefficient of variation based on the ratio of the standard deviation to the mean. For RNA-seq analyses of *F. graminearum* (Connolly et al. 2013) and *F. oxysporum* (Fokkens et al. 2018), raw reads were analyzed as described above. For *F. fujikuroi*, we used RPKM gene transcription data based on an *F. fujikuroi* specific NimbleGen microarray analysis (Studt et al. 2016). To compare gene transcription levels among the more distant species and different conditions, we normalized RPKM counts and ranked gene transcription values using the *percent_rank* function of the *dplyr* R package (Wickham et al. 2019). An in-depth description of the RNA-seq analyses is available at https://github.com/crolllab/datasets.

### Orthology and Sequence Conservation Analyses

To infer conservation across closely related and distant species, we based our analyses on protein sequences. Amino acid sequence orthology within the *Fusarium* genus was predicted using Orthofinder v2.4.0 with default parameters (Emms and Kelly 2019). The analysis included eight distinct species (for 11 genomes in total) for the FGSC and 15 additional species of the genus. Protein conservation was assessed as the mean of the pair-wise amino acid sequence identity based on the top BLASTP (Camacho et al. 2009) hit between protein sequences of *F. graminearum* (PH-1) against the 25 other *Fusarium* genomes. We classified the underlying amino acid sequence as highly conserved if an ortholog of the *F. graminearum* reference genome was conserved in all of the 26 genomes. Only single-copy genes in the FGSC were retained. Conversely, orthologs failing the above criteria were classified as variable. To infer orthology and duplication events among more distantly related fungi, we analyzed the following set of genomes using Orthofinder with default parameters: *F. graminearum, F. oxysporum, N.crassa*, *M.oryzae*, *Z.tritici*, and *Scerevisiae* (Supplementary table S1, Supplementary Material online). The outline of the data sets used in the study based on orthology conservation is available in figure 1*B*. To infer the age of highly conserved genes in *Fusarium*, we used the *F. oxysporum* gene set available from the Geneorigin database covering 565 species from Ensembl and Ensembl Genomes databases following the protein family-based pipeline based on the Wagner parsimony algorithm (Tong et al. 2020). Data set and sequence accessions are listed in supplementary table S1, Supplementary Material online.

### Chromatin Immunoprecipitation Sequencing

*Fusarium* *graminearum* PH-1 strain ChIP-seq and RNA-seq data sets of the histone modifications H3K27me3, H3K36me3, H3K4m3, and H3K4m2 from fungal mycelium grown in two different culture conditions (low and high nitrogen medium) were retrieved from the NCBI SRA database (accession PRJNA221153; Connolly et al. 2013, last accessed May 21, 2020). Chip-seq raw reads were trimmed with Trimmomatic v. 032 (Bolger et al. 2014) and mapped to the *F. graminearum* reference genome using Bowtie2 v. 2.4.0 (Langmead and Salzberg 2012). Alignment bam files were converted using BEDtools v.2.30.0 (Quinlan 2014) and peak calling was performed using the makeTagDirectory and findPeak programs included in Homer v.4.11 (Heinz et al. 2010). Peak calls from replicates were merged with BEDtools intersect. Peak coverage was calculated with BEDtools coverage. Similarly, gene coverage was analyzed with BEDtools intersect based on the *F. graminearum* reference genome annotation. The peak intensity analyses nearby TSSs were performed using the deeptools package (Ramírez et al. 2016). ChIP-seq data sets for different species *F. oxysporum* (Fokkens et al. 2018), *F. fujikuroi* (Studt et al. 2016), *M. oryzae* (Zhang et al. 2021), *Z. tritici* (Feurtey et al. 2020), and *N. crassa* (Jamieson et al. 2016) were analyzed following the same procedure as described above the matching reference genome (supplementary table S1, Supplementary Material online). We identified a strongly bimodal distribution of H3K27me3 ChIP-seq derived reads on the gene body and, hence, split genes having either >50% or <50% of their coding sequence covered by H3K27me3 marks.

### Functional Enrichment Analyses and Codon Usage

GO term enrichment analyses were performed using the Fisher’s exact test based on gene counts with the *topGO* R package (Alexa and Rahnenführer 2009) and plotted using the *GOplot* R package (Walter et al. 2015). CAI between marked and unmarked and genes was calculated using the CAI software of EMBOSS (http://www.ch.embnet.org/EMBOSS/) package. We used the codon usage table for *F. graminearum* (PH-1) available from the Kazusa DNA Research Institute (Nakamura et al. 2000). Hydrophobicity (GRAVY) and aromatic scores were calculated using the CodonW software v.1.4.4 (Peden 2000). The GRAVY score represents the average hydrophobic index across all amino acids of a predicted protein. The aromatic score was calculated as the proportion of aromatic amino acids. IDR consensus predictions were performed using the Metapredict software v.1.4 (Emenecker et al. 2021). Protein IDR scores were calculated as the mean of the disorder score consensus per protein sequence. Residue IDR scores were calculated as the mean disorder score consensus per amino acid residues along protein sequences.

### RIP Mutations and Data Analyses

To detect signatures of RIP mutations, we used the software The RIPper (Van Wyk et al. 2019) on the *F. graminearum* (PH-1) genome with a window size of 1,000 bp and a 500 bp step size. Spearman rank correlation tests were visualized using the *corrplot* R package (Wei et al. 2017). Genome-wide transcription heatmaps were generated using the *pheatmap* R package (Kolde and Kolde 2015). Upset diagrams were created with the *UpSetR* R package (Conway et al. 2017). Other figures were produced using the *ggplot2* R package (Wickham 2011).

### Transcription and Protein Conservation Analyses

To investigate the association of H3K27me3 marks with protein conservation and transcription robustness, we performed linear regression modeling. We performed the analysis using the R environment (R Core Team 2020). We first log-transformed transcription variation values for normalization. We analyzed the marked (810 out of 6,070) and unmarked ortholog sets (5,260 out of 6,070) of the highly conserved gene data set separately as linear regressions.

### Gene Dispensability Analysis

To infer functional dispensability of *Fusarium* genes in general, we used publicly available mutant screens for effects on host infection. For *F. graminearum*, we used the curated PHI-base (supplementary table S2, Supplementary Material online). For *F. oxysporum*, we used a mutant screening by Michielse et al. (2009).

## Supplementary Material

Supplementary data are available at *Molecular Biology and Evolution* online.

## Supplementary Material

msab323_Supplementary_Data
