# Supplementary material for: Histone H3K27 Methylation Perturbs Transcriptional Robustness and Underpins Dispensability of Highly Conserved Genes in Fungi
Source: Mol Biol Evol. 2021 Nov 9;39(1):msab323. doi: 10.1093/molbev/msab323 (PMC8789075; doi:10.1093/molbev/msab323)
Supplement: msab323_Supplementary_Data [file msab323_supplementary_data.zip › Supplementary_figure_S2.pdf]

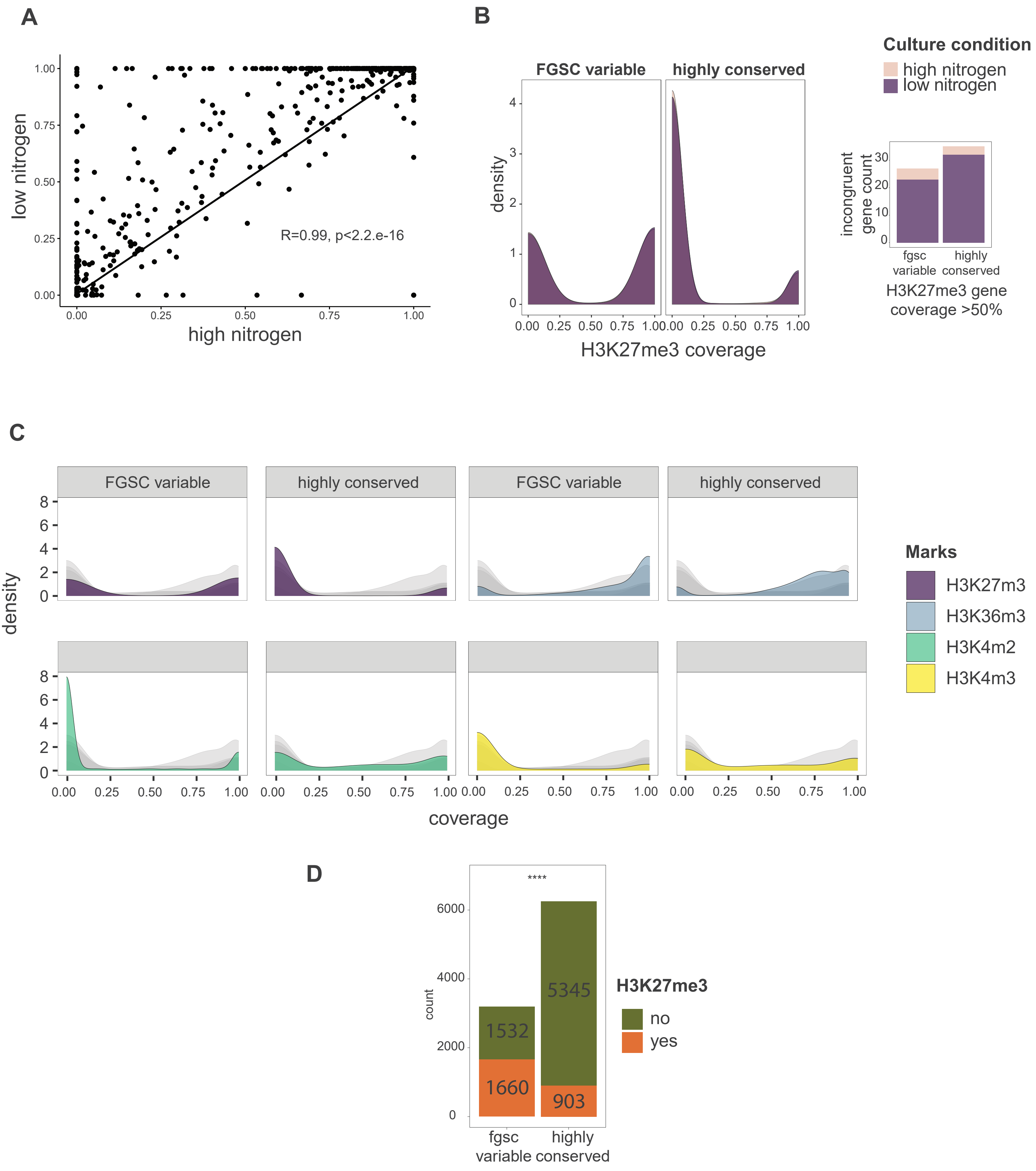

Supplementary Figure S2: Histone modification marks coverage of highly conserved and variable genes in *F. graminearum* (PH1) (Connolly et al. 2013). A) Correlation plot between H3K27me3 gene body coverage of *F. graminearum* in high nitrogen and low nitrogen culture media. A test was performed between paired samples using a Spearman correlation. B) Density plot of histone modification mark H3k27me3 in high nitrogen and low nitrogen culture media. The boxplot shows the gene count of H3K27me3 coverage incongruency between conditions. C) Density plot of *F. graminearum* marks. D) Proportion ofgenes marked by H3K27me3 in variable and highly conserved genes. Two-sample test for equality of proportions with continuity correction. p <0.0001.
