# Supplementary material for: Histone H3K27 Methylation Perturbs Transcriptional Robustness and Underpins Dispensability of Highly Conserved Genes in Fungi
Source: Mol Biol Evol. 2021 Nov 9;39(1):msab323. doi: 10.1093/molbev/msab323 (PMC8789075; doi:10.1093/molbev/msab323)
Supplement: msab323_Supplementary_Data [file msab323_supplementary_data.zip › Supplementary_figure_S3.pdf]

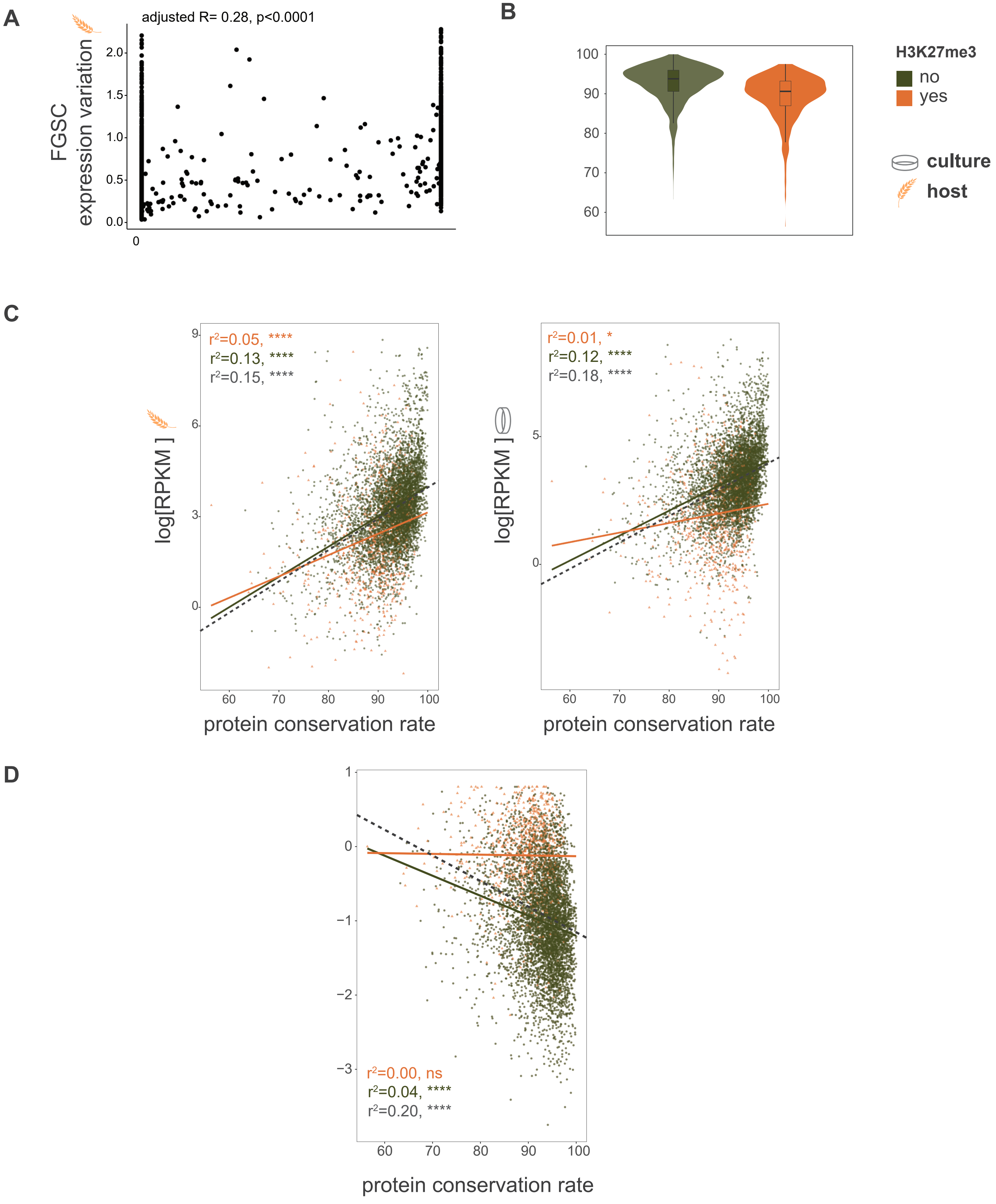

Supplementary Figure 3S: A) Spearman correlation between expression variation and H3K27me3 coverage. Adjusted r value refers to the correlation controlling for H3K4me3 marks. B) Distribution of protein conservation rate of highly conserved genes (n=6070). C) Association between gene transcription in different growth conditions (host and culture) and protein conservation rate. D) Association between transcription variation in culture and protein conservation rate. Grey r^2 value refers to the multiple regression model explaining gene expression (C) or genetranscription variation (D) based on protein conservation rate and H3K27me3 marks. The dashed grey line refers to a linear regression for the full dataset (highly conserved). Colored lines and r^2 values, refer to the linear regression of H3K27me3 unmarked (green, n=5260) and marked genes (orange, n=810). D) ns: p-value>0.05, *: p-value<0.05,**** :p-value<0.0001.
