# Supplementary material for: Histone H3K27 Methylation Perturbs Transcriptional Robustness and Underpins Dispensability of Highly Conserved Genes in Fungi
Source: Mol Biol Evol. 2021 Nov 9;39(1):msab323. doi: 10.1093/molbev/msab323 (PMC8789075; doi:10.1093/molbev/msab323)
Supplement: msab323_Supplementary_Data [file msab323_supplementary_data.zip › Supplementary_figure_S4.pdf]

A

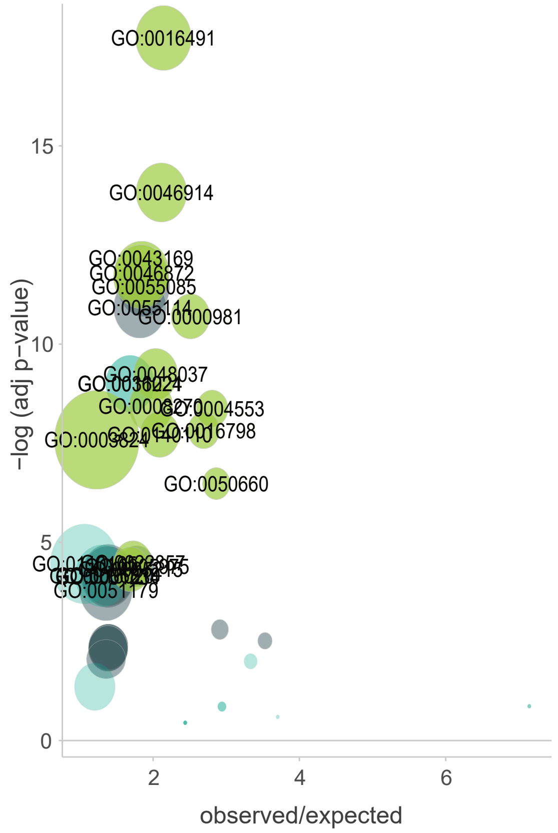

ID	Description
GO:0055085	transmembrane transport
GO:0055114	oxidation−reduction process
GO:0005975	carbohydrate metabolic process
GO:0006810	transport
GO:0051234	establishment of localization
GO:0051179	localization
GO:0016021	integral component of membrane
GO:0031224	intrinsic component of membrane
GO:0110165	cellular anatomical entity
GO:0016020	membrane
GO:0016491	oxidoreductase activity
GO:0046914	transition metal ion binding
GO:0046872	metal ion binding
GO:0043169	cation binding
GO:0000981	DNA−binding transcription factor activity
GO:0048037	cofactor binding
GO:0008270	zinc ion binding
GO:0004553	hydrolase activity, hydrolyzing O−glycosyl compounds
GO:0016798	hydrolase activity, acting on glycosyl bonds
GO:0140110	transcription regulator activity
GO:0003824	catalytic activity
GO:0050660	flavin adenine dinucleotide binding
GO:0022857	transmembrane transporter activity
GO:0005215	transporter activity

B

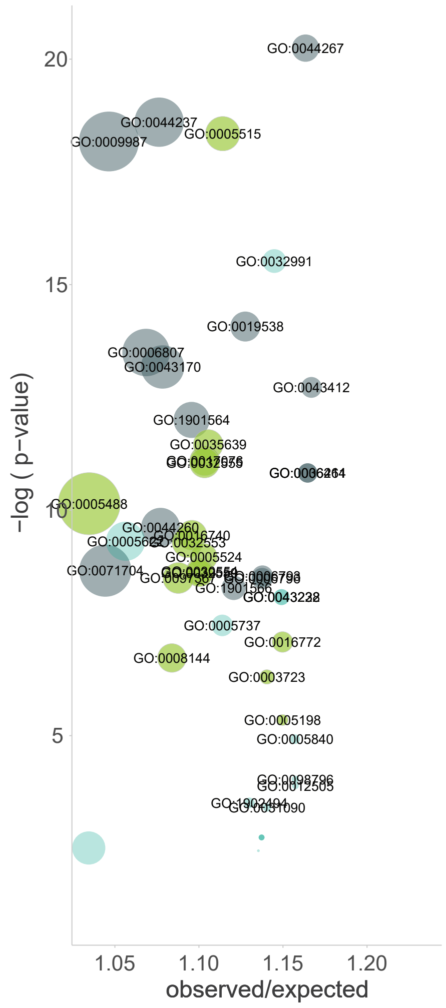

ID	Description
GO:0044267	cellular protein metabolic process
GO:0044237	cellular metabolic process
GO:0009987	cellular process
GO:0019538	protein metabolic process
GO:0006807	nitrogen compound metabolic process
GO:0043170	macromolecule metabolic process
GO:0043412	macromolecule modification
GO:1901564	organonitrogen compound metabolic process
GO:0006464	cellular protein modification process
GO:0036211	protein modification process
GO:0044260	cellular macromolecule metabolic process
GO:0071704	organic substance metabolic process
GO:0006793	phosphorus metabolic process
GO:0006796	phosphate−containing compound metabolic process
GO:1901566	organonitrogen compound biosynthetic process
GO:0032991	protein−containing complex
GO:0005622	intracellular
GO:0043228	non−membrane−bounded organelle
GO:0043232	intracellular non−membrane−bounded organalle
GO:0005737	cytoplasm
GO:0005840	ribosome
GO:0098796	membrane protein complex
GO:0012505	endomembrane system
GO:1902494	catalytic complex
GO:0031090	organelle membrane
GO:0005515	protein binding
GO:0035639	purine ribonucleoside triphosphate binding
GO:0017076	purine nucleotide binding
GO:0032555	purine ribonucleotide binding
GO:0005488	binding
GO:0016740	transferase activity
GO:0032553	ribonucleotide binding
GO:0005524	ATP binding
GO:0030554	adenyl nucleotide binding
GO:0032559	adenyl ribonucleotide binding
GO:0097367	carbohydrate derivative binding
GO:0016772	transferase activity, transferring phosphorus−containing groups
GO:0008144	drug binding
GO:0003723	RNA binding
GO:0005198	structural molecule activity

Supplementary Figure S4. Enrichment of gene ontology (GO) terms in highly conserved genes marked (A) and unmarked (B) by H3K27me3. GO term enrichment was analyzed by Fisher tests with a treshold of $p < 0.001$.
