# Supplementary material for: Histone H3K27 Methylation Perturbs Transcriptional Robustness and Underpins Dispensability of Highly Conserved Genes in Fungi
Source: Mol Biol Evol. 2021 Nov 9;39(1):msab323. doi: 10.1093/molbev/msab323 (PMC8789075; doi:10.1093/molbev/msab323)
Supplement: msab323_Supplementary_Data [file msab323_supplementary_data.zip › Supplementary_figure_S5.pdf]

A

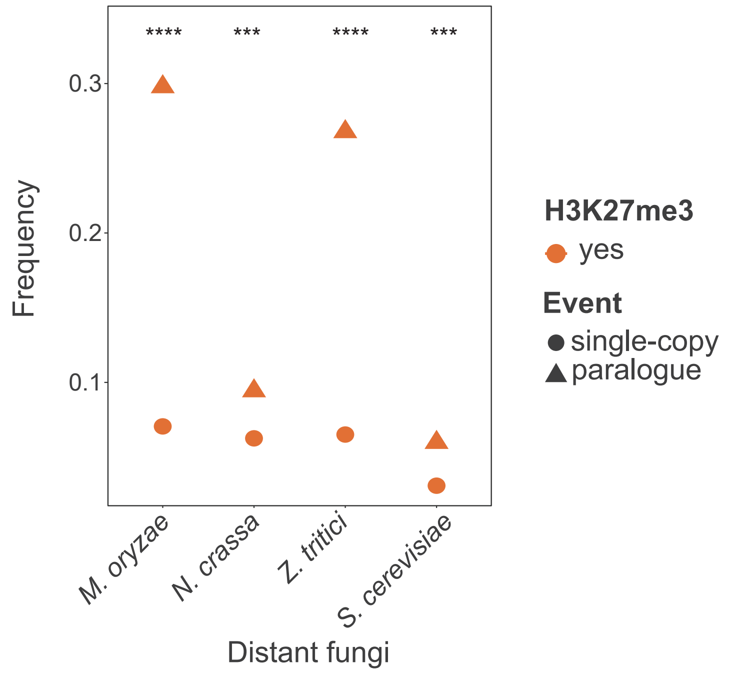

B

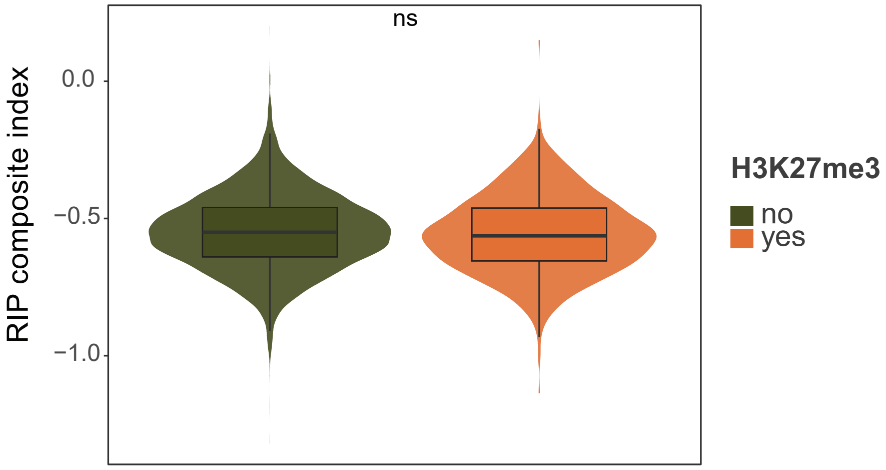

Supplementary Figure 5S: A) Proportion of H3K27me3 marked genes identified as paralogues or single-copy among the highly conserved genes (n = 810). Two-proportion z-tests were performed. B) Repeat induced point mutations (RIP) composite index in highly conserved genes marked and unmarked by H3K27me3. Wilcoxon two-sided test. ns: p > 0.05, *: p <= 0.05, **: p <= 0.01, ***: p <= 0.001, ****: p <= 0.0001.
