# Supplementary material for: Histone H3K27 Methylation Perturbs Transcriptional Robustness and Underpins Dispensability of Highly Conserved Genes in Fungi
Source: Mol Biol Evol. 2021 Nov 9;39(1):msab323. doi: 10.1093/molbev/msab323 (PMC8789075; doi:10.1093/molbev/msab323)
Supplement: msab323_Supplementary_Data [file msab323_supplementary_data.zip › Supplementary_figure_S6.pdf]

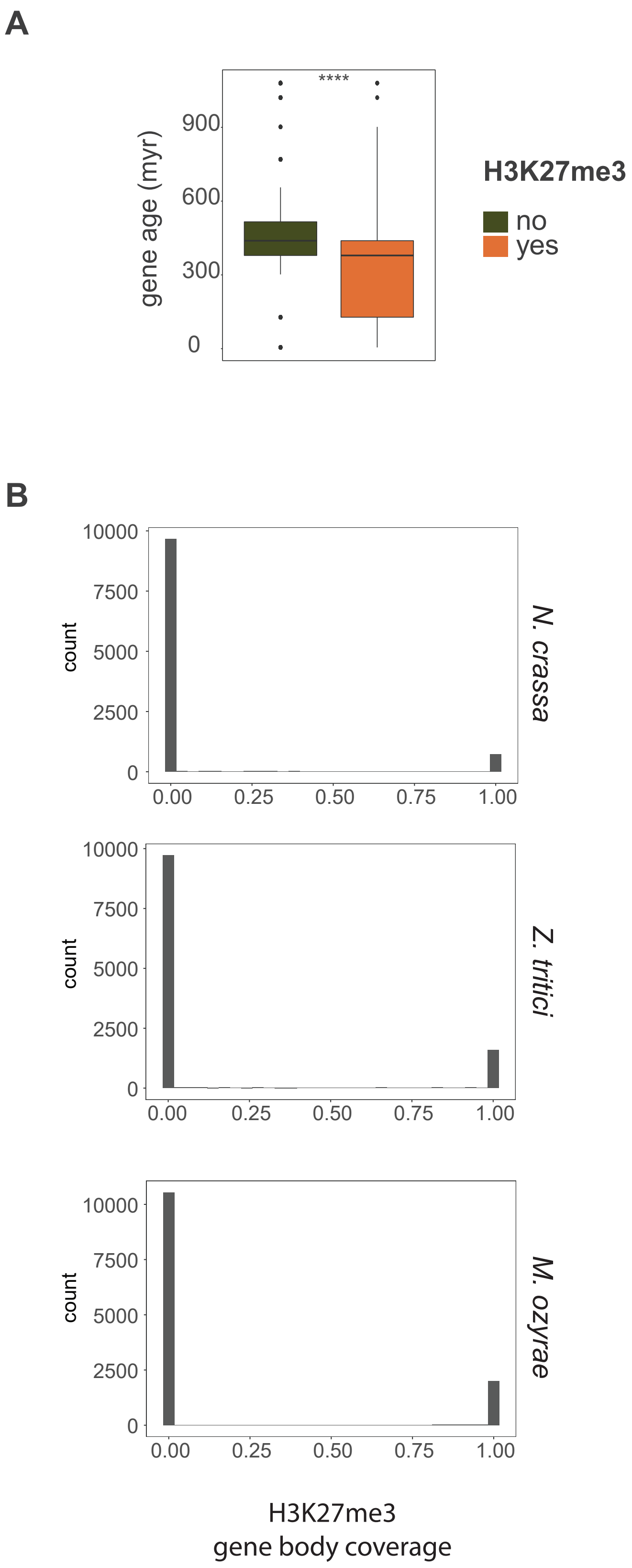

Supplementary Figure S6: A) Age inference of the highly conserved gene set. B) H3K27me3 gene body coverage distribution of distant ascomycete orthogroups. Gene coverage above or below 0.5 were defined as marked/unmarked by H3K27me3.
