# Supplementary material for: Histone H3K27 Methylation Perturbs Transcriptional Robustness and Underpins Dispensability of Highly Conserved Genes in Fungi
Source: Mol Biol Evol. 2021 Nov 9;39(1):msab323. doi: 10.1093/molbev/msab323 (PMC8789075; doi:10.1093/molbev/msab323)
Supplement: msab323_Supplementary_Data [file msab323_supplementary_data.zip › Supplementary_figure_S7.pdf]

A

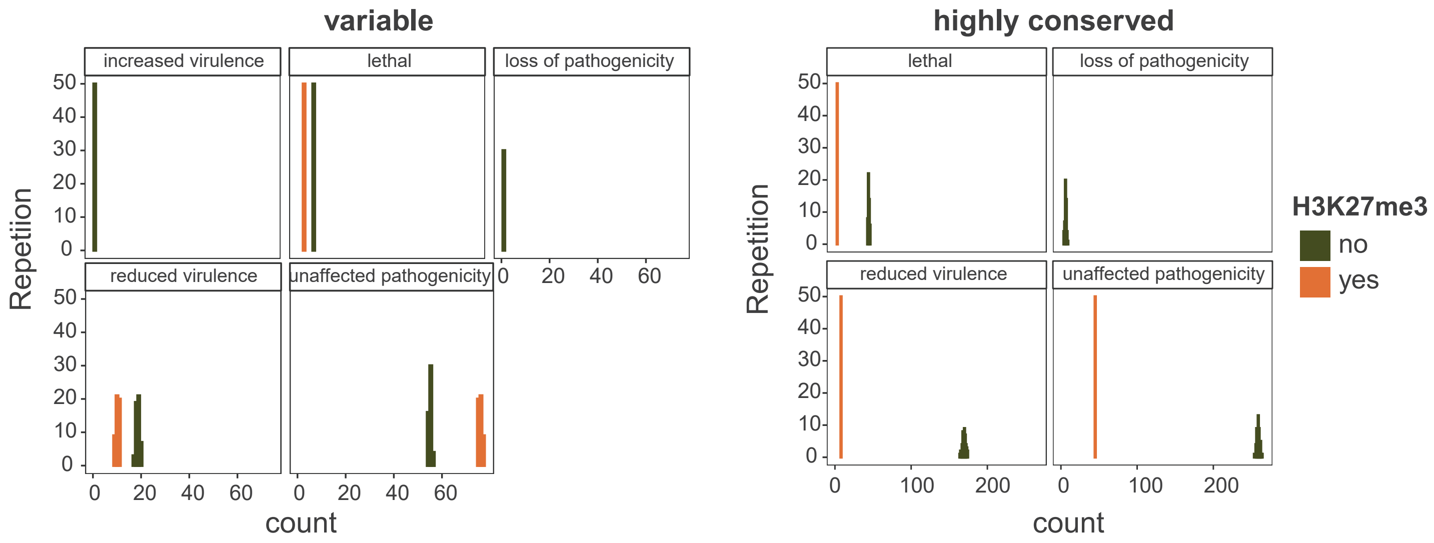

B

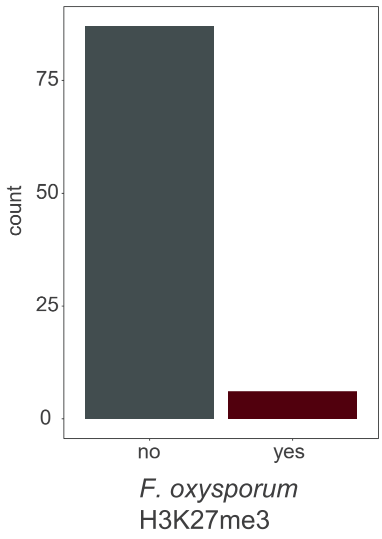

Suplementary Figure 6S: A) Distribution of phenotype count in variable and highly conserved genes after 50 iterations of duplicated targets (gene ID). Orange and green refers to H3K27me3 marks in *F. graminearum* B) *F. oxysporum f. sp. lycopersici* deletion mutants screened for loss of reduction of pathogenicity. Genes are categorized into marked and unmarked by H3K27me3 in *Fusarium oxysporum f. sp. lycopersici* ChIP-Seq (acession GSE121283).
