# Supplementary material for: Histone H3K27 Methylation Perturbs Transcriptional Robustness and Underpins Dispensability of Highly Conserved Genes in Fungi
Source: Mol Biol Evol. 2021 Nov 9;39(1):msab323. doi: 10.1093/molbev/msab323 (PMC8789075; doi:10.1093/molbev/msab323)
Supplement: msab323_Supplementary_Data [file Supplementary_figure_S1.pdf › Supplementary_figure_S1.pdf]

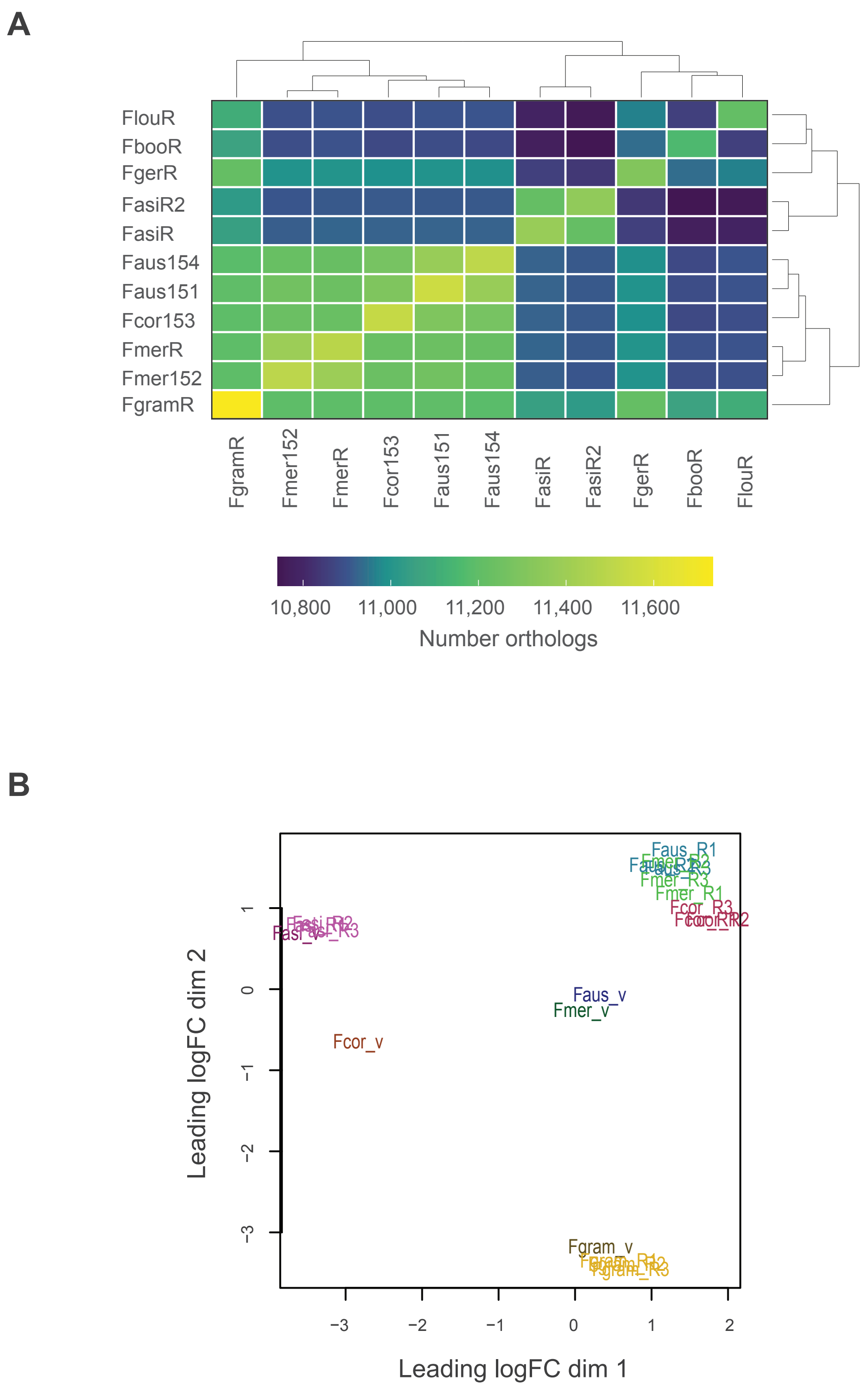

Supplementary Figure S1: A) Number of orthologs shared within the *Fusarium graminearum* species complex (FGSC). B) Multidimensional scaling analysis of transcriptomic datasets of the FGSC. Samples with suffix _v refer to culture condition replicates, _R to host infection, followed by replicate numbers. Fgram refers to *F. graminearum,* Fcor to *F. cortaderiae*, Faus to *F. austroamericanum*, Fmer to *F. meridionale* and Fasi to *F. asiatium.*
